# Indian transplant registry

**DOI:** 10.4103/0970-1591.33724

**Published:** 2007

**Authors:** Sunil Shroff

**Affiliations:** Department of Urology and Renal Transplantation, Sri Ramachandra Medical College and Research Institute, Porur, Chennai - 600 116, India. E-mail: sshroff@vsnl.com

**Keywords:** Indian society of organ transplantation, Indian Society of Organ Transplantation, registry, transplant registry, transplantation

## Abstract

An ‘Indian transplant registry’ has been established over the past two years due to the efforts of the Indian Society of Organ Transplantation. This society is about 20 years old with over 450 members who are doctors and basic scientist. The registry is currently in the first phase of its development and can be partly viewed at www.transplantindia.com. The endeavor has been undertaken with the objective of having a centralized repository of information of the various transplants that are being undertaken in India. In its first phase of the registry ‘Fast Fact’ retrospective short datasets are being captured that include the essential details of the transplant programme. The fast fact data includes the number of transplant done yearly, the sex ratio and type of transplant. So far thirteen major institutional data has been entered in the registry. In the second phase of the registry, over twenty fields are likely to be captured and all member institutions would be encouraged to enter the data prospectively. In the third phase data would be derived with ongoing audit features.. The society and its members have supported the formation of the registry and are enthusiastic about its potential.

An ‘Indian Transplant Registry’ has been established over the past two years due to the efforts of the Indian Society of Organ Transplantation (ISOT). This society is about 20 years old with over 450 members who are doctors and basic scientists. The registry is currently in the first phase of its development and can be partly viewed at www.transplantindia.com. The endeavor has been undertaken with the objective of having a centralized repository of information of the various transplants that are being undertaken in India. In the first phase of the registry ‘Fast Fact’ retrospective short data-sets are being captured that include the essential details of the transplant program. The fast fact data includes the number of transplants done yearly, the sex ratio and type of transplant. So far the data of kidney transplants of 13 major hospitals and of liver transplants of three has been entered in the registry. In the second phase of the registry, over 20 fields are likely to be captured and all member institutions would be encouraged to enter the data prospectively. In the third phase data would be derived with ongoing audit features. The society and its members have supported the formation of the registry and are enthusiastic about its potential.

## INDIAN TRANSPLANT REGISTRY

An ‘Indian Transplant Registry’ has been established over the past two years due to the efforts of the Indian Society of Organ Transplantation (ISOT). The Indian solid organ transplant program was started in the early 1970s. Since its inception, the program that started in a few select institutions in the 1970s, rapidly became popular and is at present practiced in over 100 hospitals in all the major cities of the country. Currently it is estimated that over 3000 kidneys, 100 livers and less than 10 heart transplants are done every year. These are only approximate figures as there is no central repository that collects the data from various centers. With the growing number of centers and the number of transplants that are being done in India, the ISOT has over the past few years, felt an urgent need to capture the information related to transplants and establish an Indian registry on similar lines as other well-established registries from Europe, Australia and USA.[1,2] These web-based registries are able to seamlessly integrate data of various organ transplants from different regions and the information is readily available at any given time. Some of the transplant registries, for example on pancreas and bone marrow have established successful international registries.[3,4]

Currently there are a few national registries that have been used. One of them is the National Cancer Registry Program that has been promoted by the Indian Medical Council of Medical Research with the help of the World Health Organization.[5] The chronic kidney diseases national registry in India has data of over 20,000 patients.[6] For this registry the country has been divided into five zones - North, South, East, West and Central and there are two members taken into the core group to facilitate the process of the registry. A similar effort is required to push forward the transplant registry. Transplant national registries are not available from developing countries and this is the first such effort. The Indian Transplant Registry as it evolves aims at capturing all the essential information related to transplants from various hospitals in India though the use of the web-based technology.

## EARLY YEARS OF ‘INDIAN TRANSPLANT REGISTRY’

The project of the Registry was discussed during the deliberations of the ISOT committee and general body meetings over the past five years. The unanimity on the lack of national transplant-related scientific data like the immunosuppressive regimes used, the short and long-term results of the transplants in the country gave consensus to the idea and the desire to implement the registry project.

The initial efforts in this direction were undertaken by clinicians from the Sanjay Gandhi Institute of Medical Sciences, Lucknow and were headed by Dr. Anant Kumar. The project had to be aborted after a couple of years due to the cost involvement and lack of long-term commitment on the funds required. A second attempt was made about three years ago with an initial grant of Rs. 25,000 and the author was given the responsibility to give initial shape to the registry with promises to provide further financial support for the project.

The initial objectives were:

1. Establishing an appropriate domain name for the registry.
2. Designing a software program algorithm to capture the scientific data from various hospitals.

Domain name: www.transplantindia.com was registered for a period of three years.

Computer algorithm: The biggest challenge was designing an effective algorithm flow to capture the retrospective fragmented data from various centers. After some deliberation it was decided that only limited retrospective data would be collected and the algorithm was developed on this line using a part-time senior web programmer. Prospectively 20 data fields were designed to be captured.

The registry was formally inaugurated by Dr. R.S.V. Yadav at the Jaipur annual conference of ISOT about two years ago. At present the registry has a part-time computer programmer who also substitutes as a web-master for the registry and operates from Chennai.

## OBJECTIVES OF THE NATIONAL TRANSPLANT REGISTRY

The objective of the ‘National Transplant Registry’ is to collect transplant-related scientific data from various hospitals in the country. The data so collected should at any given time be able to derive the following information:

- The total number of transplants for any organ done in the country.
- The total number of transplants done in a year.
- Essential demographic data of Indian patients undergoing transplants.
- The immunosuppressive regimes used by various centers.
- Short-term and long-term results of the allograft.
- Complications during management in the short term and long term.
- Graft and patient survival after transplants.
- The HLA profile of Indian patients.
- Number of living and cadaver transplants.
- Relationship in case of related transplants.
- Profile of donors.

The data collected is confidential and stored on a secure server and can only be accessed by a registered full member of the society. A member institution's medical staff on keying in the password has access to data of their own institution and has the facility to edit, update and upload the data. They are allowed to access and view the national or regional data but have no access to other institutions’ data.

The development of the registry is to take place in phases and in the first phase ‘Fast Fact’ retrospective limited data related to kidney and liver transplants is being captured.

In the second phase further expansion is planned that would have the capability to capture data of other organ transplants, along with 20 additional fields of data for the kidney and liver organ transplants [Table 1]. The follow-up data of each patient would require a unique file allotment for the patient and the program is likely to be much more complex. In the third phase data queries will become operational and audit can be carried out from the information provided.

**Table 1a:** Prospective data fields for kidney transplantation – Screen 1

Transplant patient information		
Date of surgery*		
Recipient first name*		
Recipient last name*		
Recipient age*		
Recipient sex*	Male	Female
Recipient address line 1*		
Recipient address line 2		
Recipient telephone no.		
(Country code - STD code - Phone no.)		
Donor first name*		
Donor last name*		
Donor age*		
Donor sex*	Male	Female
Donor address line 1*		
Donor address line 2		
City		
State		
Pin code		
Name of consultant*		
Blood group*		Rh
Tissue type	Recipient	Donor
	HLA-A	HLA-A
	HLA-B	HLA-B
	HLA-DR	HLA-DR

**Table 1b:** Prospective data fields for kidney transplantation – Screen 2

Immunosuppressive regimens and complications		
Induction regimen of	Cyclosporine	Dose-
Immunosuppressive drugs*	Azathioprine	Dose-
	Corticosteroid	Dose-
	Others	1.
		2.
		3.
		4.
		5.
Follow-op regimen of	Cyclosporine	Dose-
Immunosuppressive drugs*	Azathioprine	Dose-
	Corticosteroid	Dose-
	Others	1.
		2.
		3.
		4.
		5.
Complications *	Yes	No
	Graft loss	
	Ureteric leak	
	Vascular thrombosis -	
	Arterial	
	Vascular thrombosis -	
	Venous	
	No of episodes of rejection	Cellular
		Vascular
	Lymphocele	Minor
		(no open drainage)
		Major
	Other complications	1.
		2.
		3.
		4.
		5.

The current program has been developed using Visual Basic and ASP software. A desktop version is also planned on dot net platform that would be able to synchronize the desktop data to the remote server whenever the computer logs onto the web.

Currently the data from various hospitals is being sent by email to the web master and the data is entered into the program. There have not been many requests for issue of passwords from the members. This could either be due to the senior member's ignorance of the web-based protocols or due to lack of manpower to do this additional work.

## MATERIALS AND METHODS

Steps taken during design of registry: While designing the registry the following issues were considered.

- Domain name registration: The domain name was registered for an initial three years with instruction given for registration for further five years once the program is fully functional. This step was taken to ensure a continuity of the web address. One of the most common problems has been of popular domain names being taken up by ‘domain squatters’, who make money by selling the same name back to the person who first owned it at a premium price.
- Security issue: The database was co-hosted on a server. A separate server would have been ideal; however, this can be very expensive. Currently, an extra amount has been paid to ensure that security issues are not tampered. Keeping screen security issues in mind the program has a timeout option which means an idle screen logs out after a certain period of time. If wrong login or passwords are keyed in more than three times the system locks and does not allow the user to proceed.
- Registration process: Hospitals doing transplants in India can register on the website with their essential information and request for login name and password. An email will then be sent to the secretary of the society and the password can be issued within 24 to 48h.
- Navigation through pages: To make the registry user-friendly the algorithm for navigation was kept very simple. If a user moves from one page to another, the navigation scheme can be viewed on the top menu bar.
- Access to data: An institution is allowed to only access and change their own data. They are able to view collated data but have no access to data from another institution.
- Data collection: A lot of thought process was given on how to give the registry an instant start. It was decided to have two streams of data that could be keyed in; retrospective data would be in a limited format; whereas prospective data could be in more detailed format.
- Flexibility of the current registry: The modules of the current registry have been created using subsets of database. This means that more fields can be added in the future and the registry can be upscaled or migrated to another program if necessary.
- Audit of data: The collated data using in-built programming commands is capable of carrying out rigorous scientific analyses that can be accessed by the transplant community.

## RESULT OF THE FIRST PHASE OF DATA CAPTURE

### Kidney transplants

Thirteen major hospitals have so far submitted their retrospective data to the registry in the last one year [Table 2]. The first phase ‘fast fact data’ is retrospective data of the number of transplants done and has only limited fields. This data can be viewed by year on the registry [Table 3].

**Table 2:** Hospitals that have submitted kidney and liver data to the registry

Hospital name	City	State
Sanjay Gandhi Post Graduate Institute of Medical Sciences	Lucknow	Uttar Pradesh
Apollo Hospitals	Chennai	Tamil Nadu
Devaki Hospital Ltd.	Chennai	Tamil Nadu
Vijaya Health Centre, Vadapalani	Chennai	Tamil Nadu
Sri Ramachandra Medical College and Research Institute	Chennai	Tamil Nadu
CMC Hospital	Vellore	Tamil Nadu
Jaslok Hospital and Research Centre	Mumbai	Maharashtra
Manipal Hospital	Bangalore	Karnataka
Karnataka Nephrology and Transplant Institute	Bangalore	Karnataka
Muljibhai Patel Urological Hospital	Nadiad	Gujarat
Sri Ganga Ram Hospital	New Delhi	Delhi
Global Hospital	Hyderabad	Andhra Pradesh
Mahavir Hospital	Hyderabad	Andhra Pradesh

**Table 3:** Total Number of kidney Transplants - living and cadavers done in 13 hospitals from 1971 to Feb. 2006 in India

Year	Live		Cadaver		Total
			
	Male	Female	Male	Female	
2006	206	76	10	2	294
2005	409	166	13	1	589
2004	525	191	31	6	753
2003	553	163	16	5	737
2002	538	169	17	1	725
2001	618	186	14	3	821
2000	608	171	16	11	806
1999	505	137	22	1	665
1998	504	133	12	8	657
1997	450	172	19	5	646
1996	407	162	15	3	587
1995	434	131	2	0	567
1994	423	158	0	0	581
1993	345	109	0	0	454
1992	385	131	1	0	517
1991	350	129	0	0	479
1990	323	122	0	0	445
1989	274	148	0	0	422
1988	231	133	0	0	364
1987	226	87	0	0	313
1986	187	66	0	0	253
1985	106	61	0	0	167
1984	102	19	0	0	121
1983	44	9	0	0	53
1982	26	2	0	0	28
1981	24	4	0	0	28
1980	25	4	0	0	29
1979	21	3	0	0	24
1978	24	9	0	0	33
1977	15	7	0	0	22
1976	34	8	0	0	42
1975	23	1	0	0	24
1974	17	3	0	0	20
1973	14	3	0	0	17
1972	5	1	0	0	6
1971	5	0	0	0	5
Total	8986	3074	188	46	12294

### Liver transplants

Three of the four institutions that are doing liver transplants regularly have submitted their data.

### Cardiac and pancreas transplants

These transplant data are currently not available but efforts are being undertaken to obtain the same.

## DISCUSSION

Transplants of solid organs and tissues have been fully established over the last 20 years. The year 1994 was a historic year with the establishment of the “The Transplantation of Human Organs Act, 1994” (THOA). The act accepted brain death as a form of death and made it possible to undertake other solid organ transplants like heart, liver and lung transplants in India. The THOA also made commercial dealing in organs illegal. This law was enacted on guidelines provided by WHO that were based on the Resolution of the 44^th^ World Health Assembly urging Member Countries to incorporate into their laws the enabling provisions for legitimate transfer of organs by voluntary donors and to define death as “Brain Stem Death”.[7] The deceased donor program has resulted in 1,200 additional organs in the last 10 years - about 1050 kidneys and over 150 livers, hearts, lungs and pancreas.[8]

In the last six years liver transplants are being undertaken in various hospitals. Similarly, a few centers have also started a cardiac transplant program. The number of hospitals undertaking kidney transplants also seems to be increasing. With multiple solid organ transplants taking place in various hospitals and at various locations, the urgent need for a centralized registry cannot be overstated. The web technology makes remote integration of scientific data an easily achievable objective with minimum resources. One of the biggest advantages of such a web-based registry is that it makes its operation location-independent and makes it available instantly to the end-user. The responsibility of keying in the correct data is also with the end-user. The database can be programmed to give instant answers to complex scientific issues. Once fully operational the registry can help the members of the society to use their data in research publications. This data would also help provide a reference document to the health administrators and planners to earmark the required budget for the national or state transplant programs. Overall, an ongoing audit of regional or national transplant data would help in development of standardized protocols for better patient outcomes. At present from the available fast fact data it is possible to know the number of transplants done yearly in the previous years, the type of transplants being done - living or cadavers and the sex ratio of these transplants.

The cost of technology in India has come down considerably over the years and its availability is no more an issue. Most of the clinicians and hospitals have ready access to the Internet on their computers. The cost of connectivity has come down in the last two years and is continuing to show a further downward trend. The connectivity available is on broadband and it has also been proposed by the government to provide free broadband connection through a wireless mode in the country by the year 2009.

The initial data submission from some of the major hospitals in India has been enthusiastic; however from other centers there has been either a lack of interest or some have needed repeated reminders before the submission was made. Thirteen hospitals have submitted their kidney transplant data and these include some of the major hospitals that have a long history of transplant programs. The current total number of kidney transplants that have been recorded in the registry are 12,294 from 1971 to 2006. These also include 12060 living and 234 cadaver transplants. Interesting majority of the recipients have been males in a ratio of almost 3:1 (9174 males against 3120 females). Chennai seems to have done the maximum number of transplants (living 5692 and cadavers 140) followed by Vellore (living 2490 and cadaver 51).

The information on 145 liver transplants from three of the four major centers indicates that at present New Delhi has done the maximum liver transplants in the country. Since this program is young, it would be easier to keep the liver data accurately.

### Popularizing the usage of the registry with the society members

In the short and long term the success of the registry depends on the cooperation of the member hospitals. The initial awareness of the registry has been created by making presentations of the registry during the annual meeting of not only the ISOT but also at the sectional annual meetings of Urologists. Similar efforts have been made at the national meeting of liver transplants. The response from members at the meeting has been positive and the need for a registry is unanimously recognized. Most members are appreciative of the efforts of the society in this direction. Though the initial response to data submission has been slow, it is felt that with time the members would realize the value of the project and better participation is expected. International bodies have also promised help in this endeavor.

Strategies to popularize the registry have to be a constant priority for the society and its committee members. The magnitude of the task is such that a separate subunit within the society is needed with its own allocation of resources for the development and implementation process of the registry. The registry as it takes shape cannot be the responsibility of any one individual, as it is presently. This subunit should have the responsibility of regularly addressing various national and regional units of nephrologists, urologists, hepatologists, cardiologists and surgeons. To ensure that the end-user and member institutions truly benefit, a desktop version of the program is being created. The advantage of such a version would be that the end-user would get a free program to be used to collect and analyze their own data and at the same time ensure that the data is uploaded into central server, whenever the user logs onto the Internet. This synchronization would ensure a seamless and effortless integration of the desktop version of the registry with the remote central server program of the registry.

At present the hospitals participating require a part-time staff to enter the data prospectively. To streamline these efforts the society can provide initial grants and incentives for employment of such a person. Institutions that regularly keep their database updated can be rewarded to encourage other institutions.

### Funding to keep the registry development and sustaining the efforts

Funding for the registry can come from the corpus of the Indian Society of Transplantation. Other similar societies like the Urological Society of India and the Indian Society of Nephrology can also help with funding. Regular sponsorship from the pharmaceuticals would go a long way in sustaining the costs. The Chronic Kidney Disease registry started by the Indian Society of Nephrology has also been funded for the last three years by the industry. So far the financial investment in the development of the registry has been minimal. Ad hoc grants have been made for the registry. The registry now requires a fulltime web-master who can play the dual role of managing the registry and also training member institutions. Financial support is also required to keep the data on a secure dedicated server to support yearly hosting and maintenance charges and domain name re-registration charges.

In India not all hospitals have a fully functional medical records system and the follow-up data on diseases is often scanty. However, more recently there is a trend towards electronic storage of the data and hospitals from many states in the country are slowly shifting towards this format besides storing the hard copies of the record. This trend is likely to grow in the coming years and if the transplant registry matures over the next few years the data sets can be easily merged into the electronic medical records of the hospitals making the task of data entry a routine everyday affair. The readily available web technology with its relatively cost-effective and smart manpower, the growing number of transplants of various organs, the slowly rising numbers of cadaver transplants and the increase in the number of centers undertaking transplants makes the prospects of running an effective Indian transplant registry not a distant dream but an easily achievable objective.
